# Size and dose dependent effects of silver nanoparticle exposure on intestinal permeability in an in vitro model of the human gut epithelium

**DOI:** 10.1186/s12951-016-0214-9

**Published:** 2016-07-28

**Authors:** Katherine M. Williams, Kuppan Gokulan, Carl E. Cerniglia, Sangeeta Khare

**Affiliations:** Division of Microbiology, National Center for Toxicological Research, US Food and Drug Administration, 3900 NCTR Rd, Jefferson, AR 72079 USA

**Keywords:** Silver nanoparticles, Intestinal permeability, Cell junctions, Barrier function

## Abstract

**Background:**

The antimicrobial activity of silver nanoparticles (AgNP) has led to interest in their use in consumer products such as food contact materials, utensils, and storage containers. Incorporation of these materials into items intended for food processing and storage suggests that consumer use of these products could result in gastrointestinal exposure to AgNP, should the nanoparticles migrate from the product. The health impact of AgNP exposure is unknown, especially effects related to intestinal epithelial permeability and barrier function. This study examined the effects of AgNP exposure of different sizes (10, 20, 75 and 110 nm) and doses (20 and 100 µg/mL) on the permeability of T84 human colonic epithelial cells, which serve as an in vitro model of the human gut epithelium.

**Results:**

Results showed that effects of AgNP on the T84 epithelial cells were size- and dose-dependent, with the 10 nm AgNP causing the most significant changes. Changes in permeability of the epithelial cell monolayer, as measured by transepithelial electrical resistance, after exposure to 10 nm AgNP were most dramatic at the highest dose (100 µg/mL), but also observed at the lower dose (20 µg/mL). AgNP could be visualized inside cells using transmission electron microscopy and silver was detected in basal wells using inductively coupled plasma-mass spectrometry. Exposure to AgNP significantly affected the expression of genes involved in anchoring tight junctions, cellular proliferation and signaling, endocytosis, and cell–cell adhesion, with the 10 nm AgNP having the greatest effect.

**Conclusions:**

The results of this study show that small-size AgNP have significant effects on intestinal permeability in an in vitro model of the human gastrointestinal epithelium. Such effects have the potential to compromise the integrity of the intestinal epithelium and this disruption of barrier function could have health consequences for the gastrointestinal tract.

**Electronic supplementary material:**

The online version of this article (doi:10.1186/s12951-016-0214-9) contains supplementary material, which is available to authorized users.

## Background

The incorporation of engineered nanomaterials into industrial and consumer products is an emerging trend due to their unique physical and chemical properties. This is especially true for silver nanoparticles (AgNP), which have been shown to have considerable antimicrobial activity. Consumer products containing AgNP are marketed for functions such as infection control and odor reduction and currently include clothing, water purification systems, biocides, appliances, and medical products [1–7]. There is also great interest in using AgNP in food packaging and other food contact materials for benefits such as extended shelf life and foodborne pathogen control (bacterial and viral) and items such as kitchen utensils, food storage containers, cutting boards, and baby bottles that claim to contain AgNP are available internationally [4, 8–13]. AgNP have also been found to be generated by some edible decorations intended for baked goods [14]. In addition, colloidal silver solutions containing AgNP are marketed as health supplements and are available for purchase at grocery stores and through the internet [2, 12, 14, 15]. This suggests that consumer use of these products could result in exposure of the gastrointestinal (GI) system to AgNP and raises questions about the potential health effects of such use [12].

This exposure is of particular concern because current research continues to link more and more health conditions with dysfunction of the gastrointestinal tract. Natural processes can lead to increases in intestinal permeability, but permeability changes are often associated with inflammatory conditions such as autoimmune diseases and invasion of pathogenic microorganisms [16–18]. An increase in intestinal permeability often occurs through disruption of the tight junctions, proteins that link epithelial cells together into a functional barrier capable of blocking the passage of microorganisms and larger molecules, while allowing absorption of water and nutrients [16, 17]. This function is critical to a healthy gastrointestinal system and many conditions specifically have been linked to increased permeability of the gut, including Crohn’s Disease, ulcerative colitis, Type I diabetes, irritable bowel syndrome, GI infection, celiac disease, and graft-versus-host disease [16, 19]. The exact contribution of increased gut permeability to disease states is unknown, but current evidence suggests that increases in permeability may prompt cycles of intestinal inflammation, causing further increases in permeability and setting the GI tract on an inflammatory course that exacerbates disease progression [16]. As previously mentioned, the growing incorporation of AgNP into common household goods, particularly food contact materials, increases the likelihood of human intestinal exposure to such materials. Additionally, a number of studies have shown that exposure to AgNP may cause toxicity and inflammatory responses [20–23]. Therefore, there are legitimate concerns regarding whether the consumption of AgNP could lead to increased permeability of the intestinal epithelium.

The purpose of this study was to perform an in vitro evaluation of the effects of AgNP on the permeability of the intestinal epithelium as modeled by T84 cells, an immortalized human colonic epithelial cell line. These cells serve as an excellent in vitro model for the human gut epithelium because they both produce mucus and, on transwells, form polarized monolayers in which changes in permeability can be measured quantitatively [24–26]. To our knowledge, this is the first study using the T84 cell line to evaluate the potential gastrointestinal effects of silver nanomaterials. In this work, monolayers of T84 cells were inoculated with two doses (20 and 100 µg/mL) of AgNP of different sizes (10, 20, 75 and 110 nm) to evaluate changes in paracellular permeability and cell toxicity. Effects of AgNP exposure on cellular permeability were determined by a comprehensive approach examining transepithelial electrical resistance (TER), immunofluorescence, mRNA gene expression, and transmission electron microscopy (TEM). Furthermore, the ability of the silver to pass through the epithelial cell monolayer was confirmed by inductively coupled plasma-mass spectrometry (ICP-MS).

## Results

### Characterization of nanoparticles and T84 monolayers

**Fig. 1:**
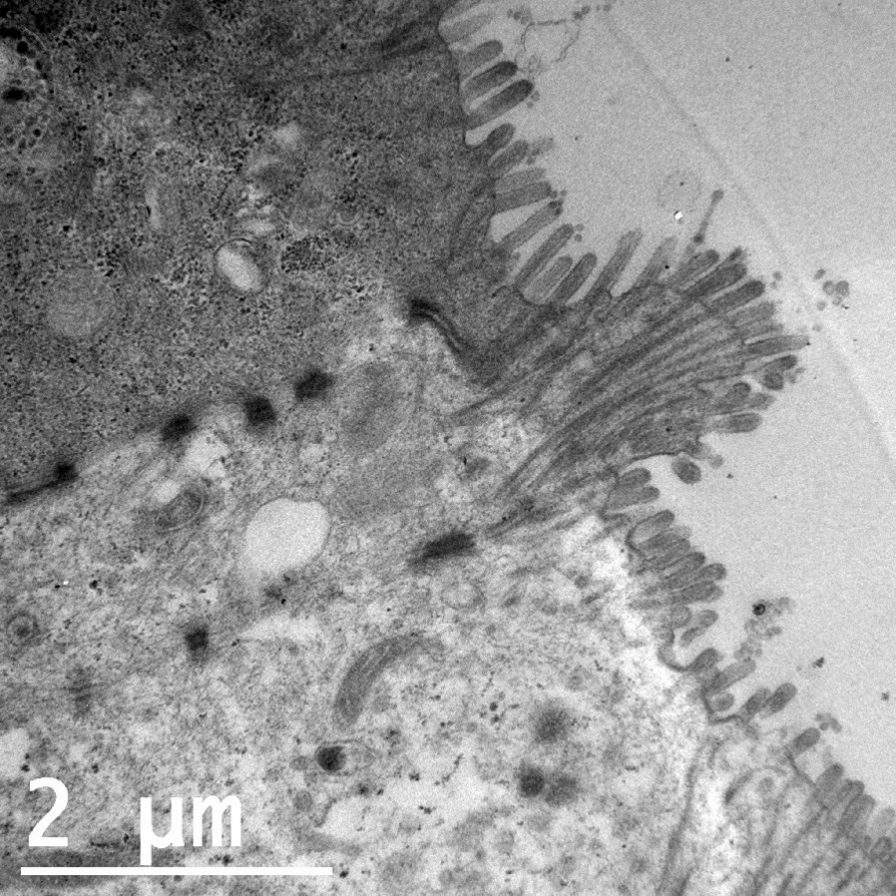
TEM image of T84 cells grown on transwells showing polarized cells with villi and junctions between individual cells

All nanoparticles were found to be sterile by plate-growth assay and below acceptable levels for endotoxin (<1 EU/mL), given the concentrations of AgNP used in this study [27, 28]. Protein corona was assessed by comparing protein gel bands from samples eluted from cell culture-incubated AgNP with bands from untreated cell culture medium. Minor protein corona formation was observed at the 100 µg/mL concentration on the smaller (10 and 20 nm) AgNP, but none was observed at the 20 µg/mL concentration (data not shown). This suggests that the 20 µg/mL AgNP concentration results in little protein adsorption and that the particles are relatively unmodified and available for biological interaction. TEM analysis of T84 cells grown on transwells (Fig. 1) showed polarized cells with villi and the presence of cell–cell junctions between individual cells, demonstrating the ability of T84 cells to form functional epithelial layers under in vitro conditions.

### Toxicity effects and cellular viability after AgNP exposure

**Fig. 2:**
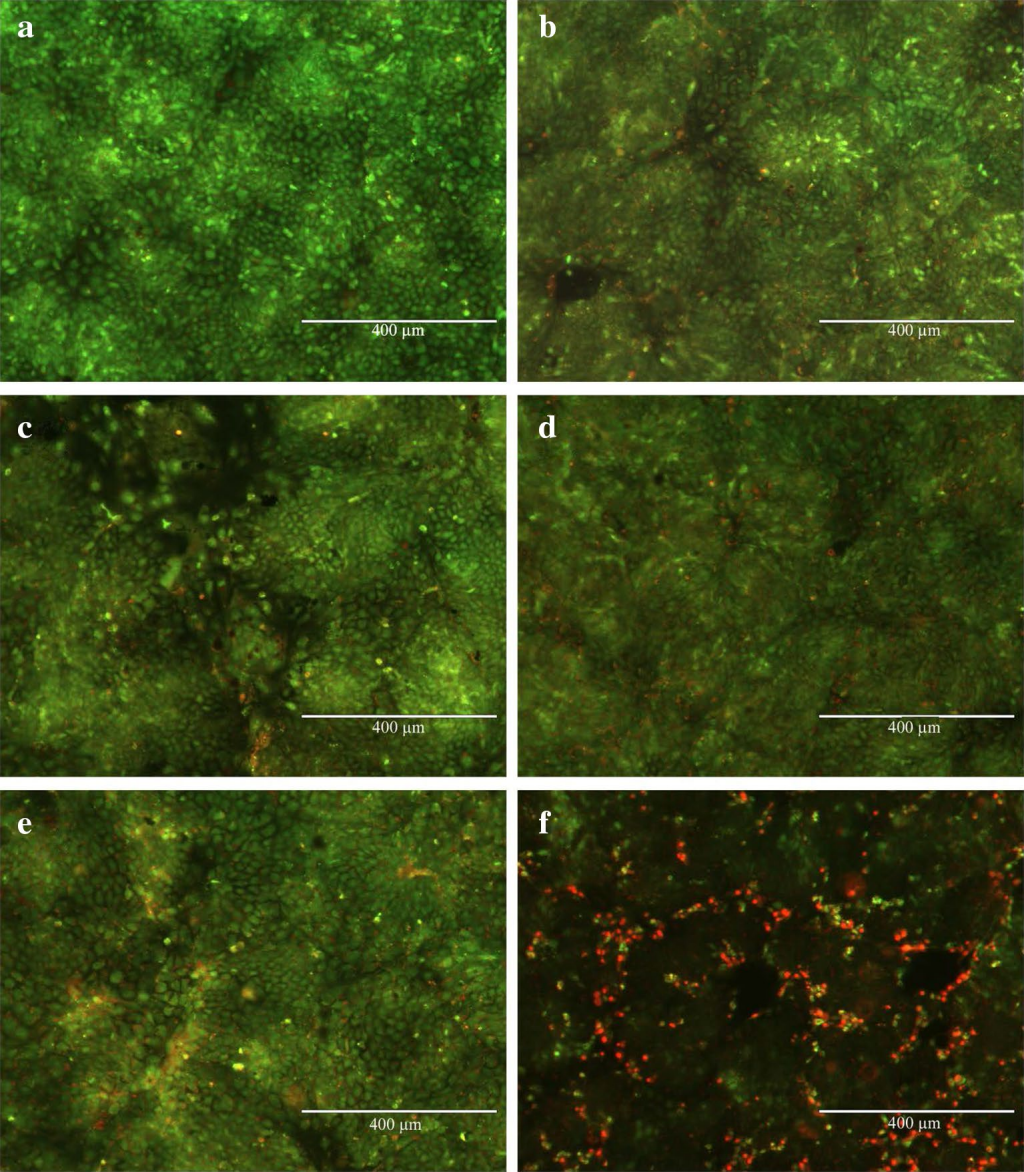
Acridine orange/ethidium bromide staining of T84 cell monolayers showing **a** control cells and cells after treatment with **b** 10 nm, **c** 20 nm, **d** 75 nm, and **e** 110 nm AgNP (100 µg/mL) for 48 h. T84 cells after treatment with 100 µg/mL of silver acetate (**f)** for 3 h are also shown

Two distinct methods were used to evaluate cellular response to AgNP exposure. Cytotoxic effects of AgNP exposure on T84 cells were evaluated by acridine orange/ethidium bromide staining. Viable cells stained by this method fluoresce green, while apoptotic and necrotic cells fluoresce orange–red. Stages of apoptosis can also be estimated from the state of chromatin in the cell nucleus [29, 30]. Treatment of cells for 48 h with AgNP of different sizes (Fig. 2b–e) showed little to no change compared to control (Fig. 2a), indicating that AgNP treatment did not induce cell death. However, after treatment with 100 µg/mL of silver acetate for 3 h, a number of dead/dying cells are visible (Fig. 2f). An image from the 3 h time point is shown because treatment for 48 h resulted in complete cell death, with very few cells still attached to the coverslip. Similarly, cells treated with 20 µg/mL of silver acetate for 48 h were almost universally in late stage of cell death (data not shown).

**Fig. 3:**
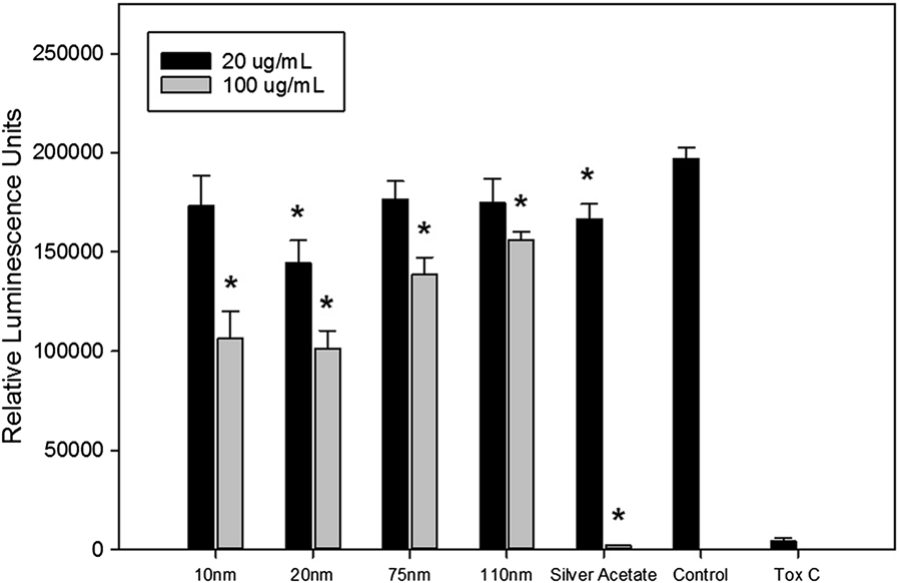
Results of ATP-based cell viability assay after treatment with AgNP, silver acetate, and controls for 48 h. ‘Control’ cells were treated only with water (negative control), while ‘Tox C’ cells were treated with 0.5 % hydrogen peroxide (positive control). *Error bars* represent SEM and presence of *asterisk* denotes significance at p < 0.05

Impact of 48 h AgNP exposure on T84 cell viability was evaluated through use of a commercially available ATP-based luminescence assay. Very little effect on cell viability was demonstrated by treatment with AgNP at the 20 µg/mL dose, with the exception of the 20 nm particle. However, significant decreases in cell viability were observed for all AgNP sizes when cells were treated with the 100 µg/mL doses (Fig. 3). Cells treated with silver acetate at the lower dose showed a small, but significant, decrease in viability, while wells treated with 100 µg/mL of silver acetate showed that almost no viable cells remained.

### Effect of AgNP exposure on epithelial permeability

**Fig. 4:**
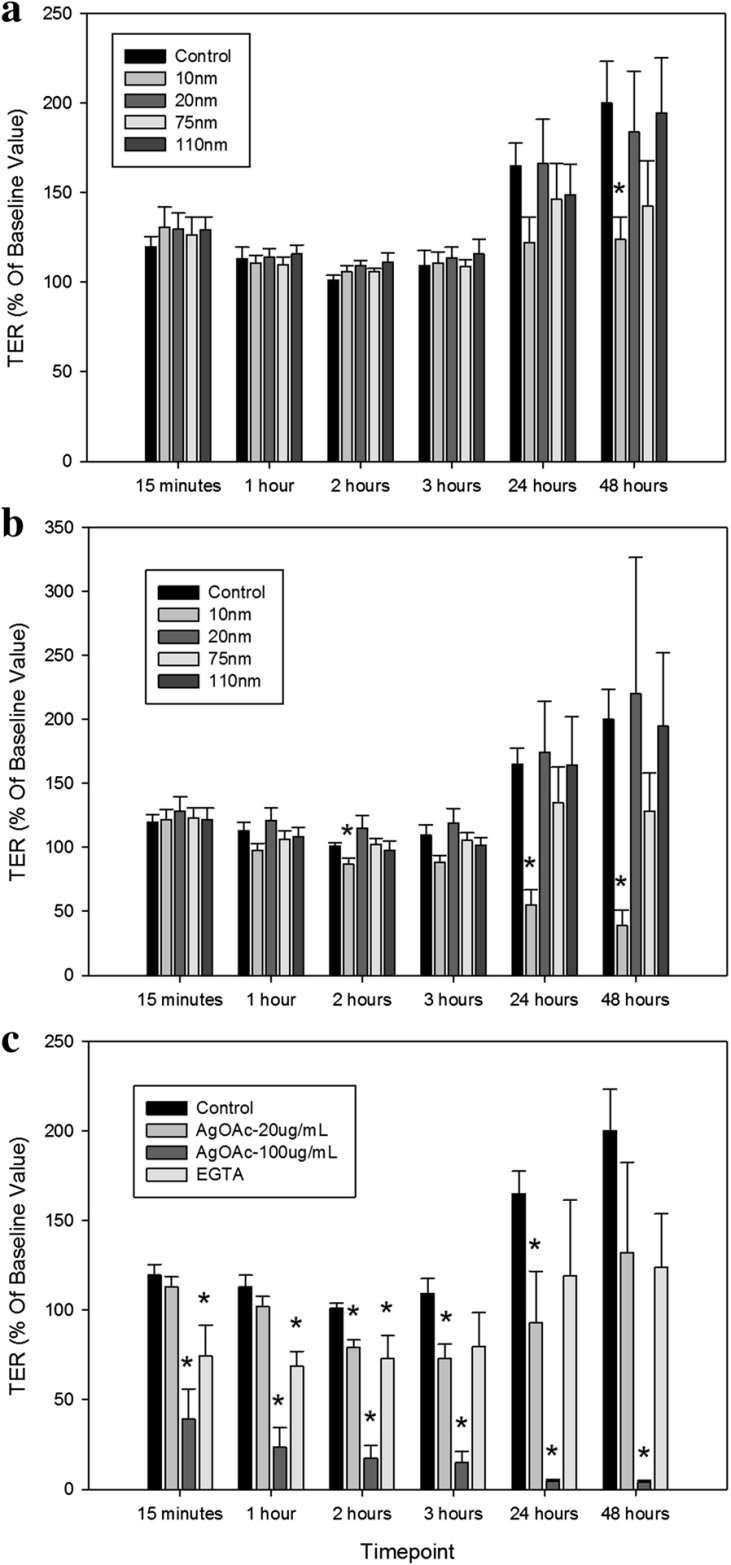
Relative changes in TER of T84 cells after exposure to AgNP-20 µg/mL (**a)** AgNP-100 µg/mL (**b)**, and controls (**c**). *Error bars* represent standard error of the mean (SEM) of 6 independent experiments

To measure changes in permeability, T84 cells grown on transwells were exposed to AgNP (10, 20, 75 and 110 nm) at doses 20 µg/mL (Fig. 4a) and 100 µg/mL (Fig. 4b) for 48 h. TER measurements to assess changes in resistance compared to initial reading were taken at 15 min, 1, 2, 3, 24 and 48 h. Silver acetate at 20 and 100 µg/mL, in addition to EGTA, were used as controls (Fig. 4c). For both 20 and 100 µg/mL doses, the only tested AgNP that resulted in significant changes in permeability was the 10 nm particle. For the 20 µg/mL dose, the 10 nm AgNP caused significant depression of TER, indicating increased permeability of the cell layer, at the 48 h time point (p = 0.01). After exposure to the 100 µg/mL dose, the changes were much more dramatic, with significant increases in permeability observed at the 2 h (p = 0.03), 24 h (p < 0.001), and 48 h (p < 0.001) time points. Cells showed increased permeability after exposure to silver acetate at both doses. Decreases in TER measurements were significant for the 20 µg/mL dose at the 2, 3 and 24 h time points. Dramatic increases in permeability were seen for the 100 µg/mL dose at all time points and TER values continued to decrease over time. Cells exposed to EGTA, a positive control capable of inducing increased permeability, showed the expected reduced TER measurements at 15 min, 1 and 2 h time points. These values gradually recovered, likely as a result of the compound passing from the apical compartment through the cell layer and becoming further diluted in basal media. Decreases in TER were also observed for 10 nm AgNP at 24 h (20 µg/mL dose) and 1 and 3 h (100 µg/mL dose) time points, but these results were not statistically significant.

### Penetration of AgNP into the epithelial cell layer

**Fig. 5:**
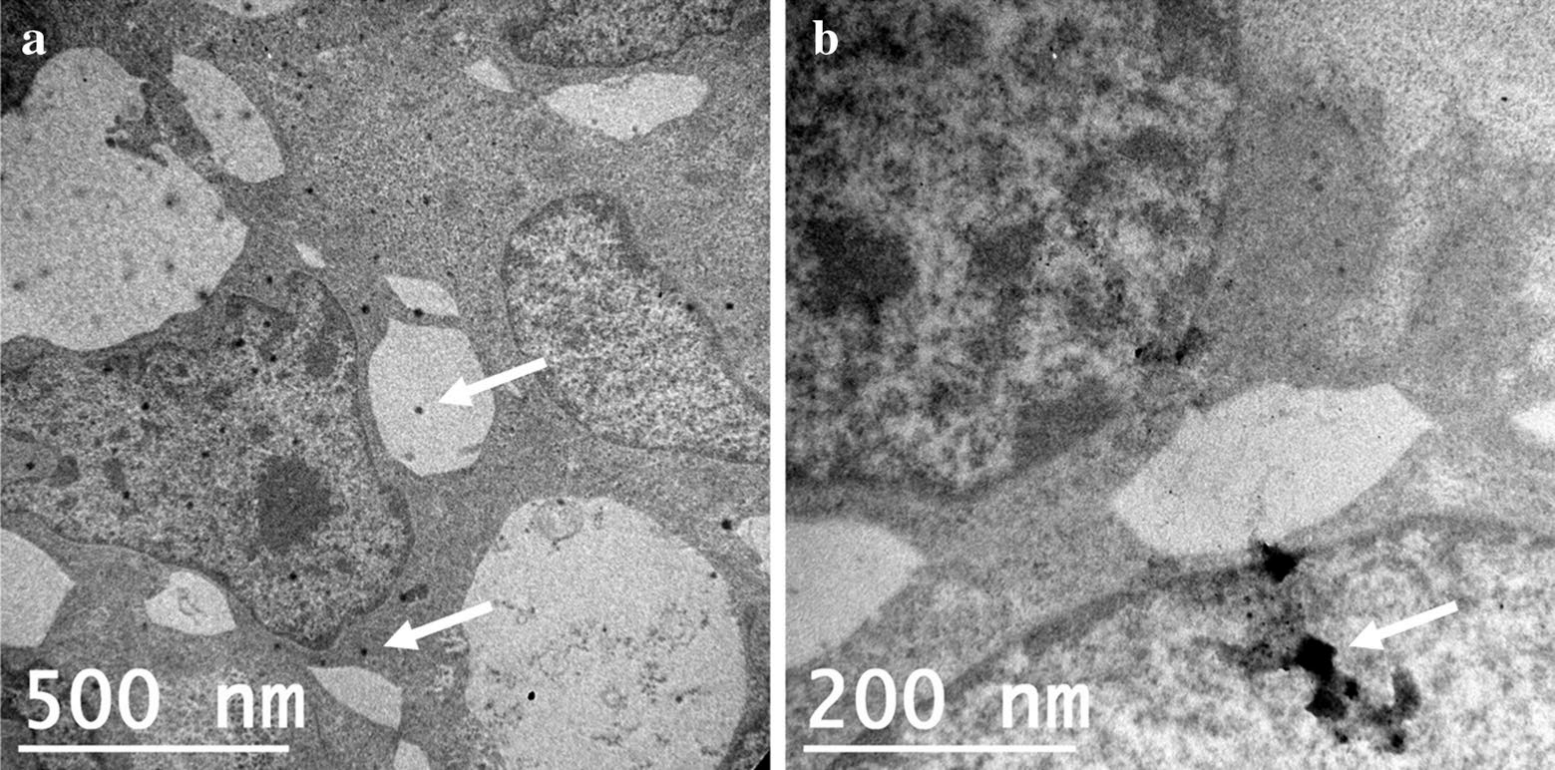
TEM images of T84 cells with internalized nanoparticles (*arrows*) after exposure to **a** 10 nm and **b** 75 nm AgNP

Given the observed effect of AgNP on epithelial layer permeability, we used transmission electron microscopy (TEM) to see if AgNP could be observed inside T84 epithelial cells after exposure. Cells were treated with AgNP (10 and 75 nm) and incubated for 48 h before fixation and imaging. Cells treated with 10 nm AgNP (50 µg/mL) (Fig. 5a) and 75 nm AgNP (20 µg/mL) (Fig. 5b) revealed potential AgNP within cells. These images showed dark particles with sizes approximating known AgNP size distributions. Analysis of these particles using energy dispersive X-ray spectroscopy (EDS) indicated the presence of silver, suggesting that these may be internalized AgNP (Additional file 1: Figure S1). Interestingly, the observed particles in the 10 nm AgNP sample do not appear to be agglomerated within the cell. Thus it appears that the small sized AgNP may gain entry into the cells in single or monomeric form. In cells treated with 100 µg/mL doses, small amounts of silver were detected in media (collected from the basal compartment of transwell) by ICP-MS, suggesting that silver was passing through the cell layer from the apical compartment. No silver was detected in basal media of cells treated with 20 µg/mL doses of AgNP and silver acetate. The detected amounts ranged between 0.82 and 1.44 % of total spiked silver for the 2 h time point and 0.71–6.06 % for the 48 h time point (Additional file 1: Table S1). However, this method does not distinguish between different forms of silver, so it is unknown if the silver detected was in the form of AgNP or silver ions.

### Effect of AgNP exposure on expression of molecules involved in barrier function

**Fig. 6:**
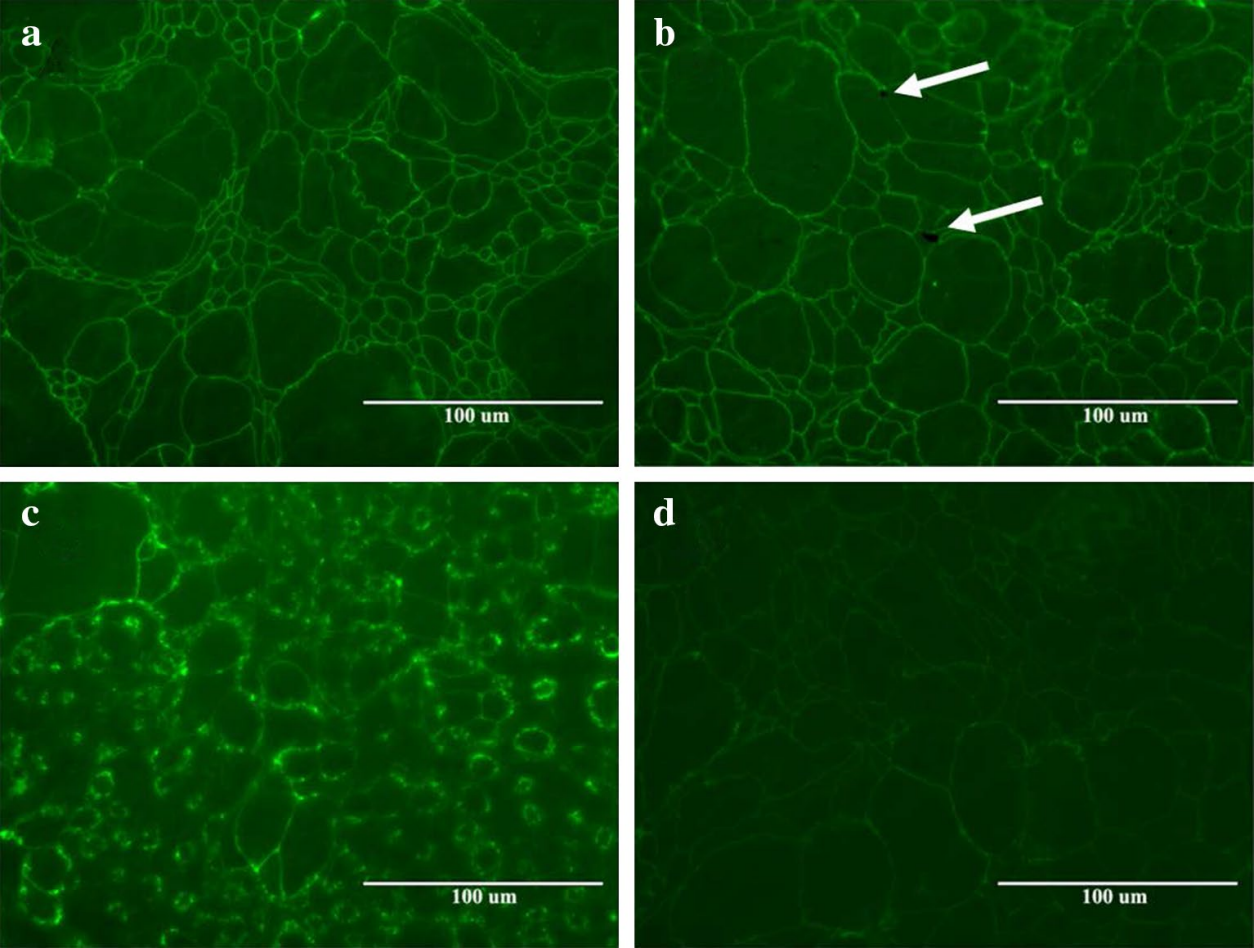
Expression of Occludin protein in T84 cells after 24 h treatment with **a** control, **b** 10 nm AgNP, **c** EGTA, and **d** silver acetate. *Arrows* point to suspected nanoparticle agglomerates in AgNP-treated cells

Immunofluorescence analysis of AgNP-treated cells was performed to determine effect on two major cellular adhesion molecules, Occludin (Fig. 6) and E-cadherin (Additional file 1: Figure S2). Occludin is one of the primary components of tight junctions and E-cadherin is an important component of adherens junctions in epithelial cells. T84 cells were treated with the 10 nm AgNP at a concentration of 100 µg/mL because this size and dose combination showed permeability increases in the TER data. Cells treated with EGTA and water were used as a positive and negative control, respectively, and cells treated with silver acetate (50 µg/mL) were used to assess the impact of silver ions. The 50 µg/mL dose was selected based on the results of the TER study, as the 20 µg/mL dose did not result in a major change in permeability at 24 h, while the 100 µg/mL dose showed drastic increases. For both Occludin and E-cadherin, no significant effects were observed after 24 h treatment with 10 nm AgNP. However, slight changes in cell–cell junctions can be seen after silver acetate treatment. For both proteins, EGTA caused major disruption of cellular adhesion. Interestingly, dark spots (Figs. 6b and S2B—shown with arrows) were consistently present in cells treated with AgNP, but not in any of the other controls, suggesting the presence of AgNP agglomerates on the monolayer surface.

### Impact of AgNP exposure on expression of human cell junction genes

**Fig. 7:**
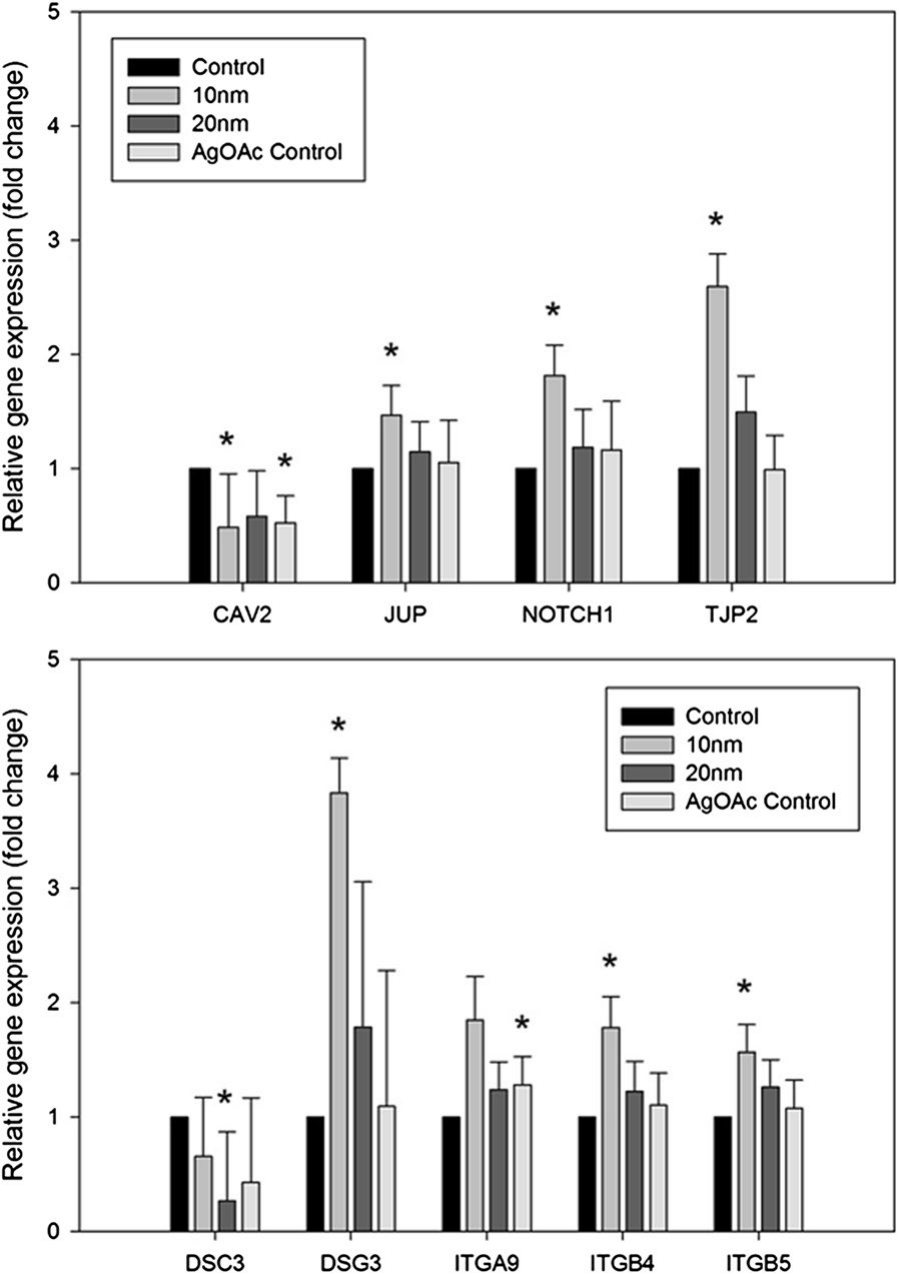
Changes in relative expression of genes related to cell–cell junctions and epithelial barrier function in T84 cells exposed to 10 nm AgNP, 20 nm AgNP, and silver acetate compared to control cells.* Error bars* represent SEM and presence of * asterisk* denotes significance at p < 0.05

T84 epithelial cell monolayers were evaluated for changes in gene expression resulting from exposure to AgNP. Effects of 48 h exposure to 10 and 20 nm AgNP (100 µg/mL) and silver acetate (20 µg/mL) were evaluated using a PCR array plate with 84 genes related to cell junctions and permeability. The 100 µg/mL dose was used for the AgNP as it was the dose at which significant effects were observed in the TER experiment, while the 20 µg/mL dose was used for the silver acetate as the cytotoxicity data showed that the 100 µg/mL dose caused toxicity to cells. The tested AgNP were also selected based on the TER experiment, as it was intended to compare changes in gene expression resulting from exposure to AgNP that both induced (10 nm) and did not induce (20 nm) significant changes in permeability. Genes with significant changes in expression were those with p < 0.05 when compared to control (Fig. 7). Of the three groups, treatment with the 10 nm AgNP showed the most significant changes in gene expression compared to treatment with the 20 nm AgNP or silver acetate. After exposure to the 10 nm AgNP, T84 cells demonstrated downregulation of the Caveolin 2 (*CAV2*) gene and upregulation of Desmoglein 3, Integrin β4, Integrin β5, Junction plakoglobin, Notch 1 and Tight junction protein 2 (*DSG3, ITGB4, ITGB5, JUP, NOTCH1,* and *TJP2)* genes. Of these genes, *DSG3* and *TJP2* showed the greatest changes in expression, with 3.8- and 2.6-fold increases, respectively. Treatment with the 20 nm AgNP only resulted in significant changes to one gene, Desmocollin 3 (*DSC3)*, which was downregulated. Exposure to silver acetate (20 µg/mL) resulted in downregulation of the CAV2 gene and upregulation of Integrin α9 (*ITGA9*). The *CAV2* gene was the only gene demonstrating significant changes in expression across more than one treatment group, as it was downregulated in both 10 nm AgNP and silver acetate groups. *CAV2* was also downregulated in the 20 nm AgNP treatment group, but the result was not significant. A number of claudin genes also appeared to be affected by treatment with AgNP and silver acetate, although the result was not statistically significant. The gene expression profile clearly indicates that the pattern of changes in permeability caused by the silver ions is distinct from that caused by the AgNP.

## Discussion

One of the most important functions of the gastrointestinal epithelium and supporting elements, such as the mucus layer, is as a physical and biochemical barrier between the external environment present in the lumen and the interior of the human body [16, 31]. As such, the state of epithelial integrity can serve as an indicator of overall gastrointestinal health. A previous study by our group found size and dose-dependent intestinal responses to AgNP in an in vivo model [32]. The present study is an in vitro approach to assess if, in fact, AgNP toxicity is size and dose dependent in intestinal epithelial cells. Based on results obtained from our earlier study, a 20 nm AgNP was added to the group of previously tested particles to further explore AgNP size-dependent effects. In this study, the most substantial effects on the intestinal epithelium were observed with the 10 nm AgNP, both in the permeability and gene expression analyses, compared to the 20, 75 and 110 nm AgNP.

In the TER experiment, changes in the intestinal cell epithelium permeability after exposure to 10 nm AgNP were seen as early as 24 h at the 20 µg/mL dose, with significant increases at the 48 h time point. Exposure at the 100 µg/mL dose caused significant increases in cellular permeability, even after exposure periods as short as 2 h. An immunofluorescence-based evaluation of Occludin and E-cadherin protein expression showed little to no change after treatment with high-dose 10 nm AgNP, suggesting that the increases in permeability observed in this study occur by a mechanism not involving these two major cellular adhesion proteins. This result correlated well with the gene expression data, which also showed minimal impact of AgNP exposure on these two genes. Observation of monolayers by TEM after exposure to AgNP showed un-agglomerated nanoparticles inside of intestinal epithelial cells, indicating that 10 and 75 nm particles are capable of penetrating the epithelial cell surface. Analysis of basal media after treatment of epithelial cell monolayers with AgNP suggests that small amounts of silver are capable of passing through the in vitro cell layer. However, more work is needed to establish how permeability changes affect the movement of silver through the cell layer and to determine whether the passaged silver is in the form of AgNP or silver ions.

In agreement with the permeability data, most significant changes in gene expression were observed after exposure to the 10 nm AgNP. Exposure to 10 nm AgNP significantly increased expression of the tight junction protein-2 (*TJP2*) gene. This gene encodes ZO-2, a protein involved in anchoring tight junctions that has been shown to inhibit the Wnt pathway and cellular proliferation and promote apoptosis [33]. Therefore, the upregulation of this gene by 10 nm AgNP exposure suggests that this gene could play a role in determining cell fate upon exposure to small AgNP. The fact that downregulation of caveolin-2 (*CAV2*), which encodes a protein integral to the formation of caveolae and involved in many other cellular processes, was strongest for silver acetate and 10 nm particles, but that similar effect was also observed for 20 nm particles, indicates that this effect may be a result of silver ion exposure during treatment with silver acetate and a cumulative effect of ions and nanoparticles during AgNP exposure. Interestingly, there is evidence that *CAV2* may play a role in the gut response to endotoxin, as CAV2-deficient mice were shown to be more sensitive to LPS exposure than wild-type mice, demonstrating more gastrointestinal tissue damage and increased intestinal permeability [34]. Increased sensitivity to LPS as a result of decreased expression of *CAV2* could result in altered interactions between host and commensal bacteria, which has been observed in a previous AgNP study in a rat model [32]. AgNP exposure also resulted in significant effect on three genes (*DSG3, DSC3,* and *JUP*) that encode elements of desmosomes, intercellular junctions that are involved in maintenance of strong adhesion between cells. Interestingly, two of these genes were significantly upregulated and one was downregulated. This could indicate that cells treated with AgNP were attempting to compensate for the increased permeability by increasing the expression of molecules involved in cell–cell adhesion. Such a mechanism could serve a protective function in vivo, serving to reinforce epithelial barrier integrity during AgNP exposure. These observed effects of 10 nm AgNP on intestinal permeability are further supported by a recently published in vivo study that found greater silver accumulation in rat tissues after oral administration of 10 nm AgNP than 75 and 110 nm particles. The authors suggested that this could be a result of 10 nm AgNP more easily penetrating the intestinal epithelial layer [35].

To evaluate whether cytotoxic effects of AgNP could be responsible for the increases in epithelial permeability observed during the TER study, cell toxicity as well as cell viability assays were utilized. We observed apparent discrepancy between the staining-based cell death assay and the luminescence-based viability assay. These two assays (staining vs luminescence) measure different markers of cell health: the staining-based assay indicated that there was little or no effect of AgNP on T84 cytotoxicity, while the ATP-based viability assay showed a marked decrease in luminescence intensity for cells treated with some AgNP at the 100 µg/mL dose. These results could indicate that the treated cells had decreased metabolic activity while remaining viable. This effect is certainly feasible, but the authors believe that this difference could also be explained by the adhesion of AgNP to the cell monolayer surface, which resulted in a dark deposit on the cells. This effect, most prominent in wells treated with small (10 and 20 nm) AgNP at the highest dose (100 µg/mL), was present even after removal of media from the wells and likely interfered with the luminescence-based assay. Similar effects have been observed for engineered nanomaterials in other assays [36]. Based on this result, staining-based assays may be more reliable methods to determine cellular toxicity effects of AgNP. The decrease in luminescence after treatment with the 10 nm AgNP at the 100 µg/mL dose appears to parallel the decrease in TER after the same treatment. However, a decrease in luminescence was also observed for the 20, 75 and 110 nm AgNP at the 100 µg/mL dose and for the 20 nm AgNP at the 20 µg/mL dose, none of which showed any significant effects in the TER study. Combined with the results of the staining-based assay, which showed little cytotoxic effect of any AgNP exposure, these observations suggest that AgNP cytotoxicity is not responsible for the observed increase in permeability. However, cells treated with silver acetate exhibited both significantly increased cell death and reduced cell viability, especially at the higher dose, indicating that the increased permeability is likely a result of cytotoxic effect.

Several groups have published in vitro cell culture-based assessments of AgNP on intestinal epithelial cell lines [37–42]. Most of these studies were performed using the Caco-2 cell line and found varying results regarding cytotoxicity and impact of AgNP on epithelial cells. However, in agreement with the results of this study, most of these works also found that impacts on cells were size- and dose-dependent, with smaller-sized particles demonstrating a larger effect. There is growing interest in developing novel in vitro approaches to mimic in vivo conditions in the GI tract. Recently, Georgantzopoulou et al. [42] used a co-culture method to incorporate mucus into a Caco-2-based system, finding that the mucus layer influenced AgNP toxicity effects and inflammatory responses in cells. As previously mentioned, one of the unique characteristics of T84 cells is that they produce a mucus layer [43], which can be visualized through Periodic acid-Schiff staining of cell monolayers (Additional file 1: Figure S3). Throughout experimentation, our group observed the presence of suspected AgNP agglomerates on the cell monolayer surface when viewed under a microscope. This is unsurprising, as it is well-documented that mucus binds and traps nanoparticles, especially those that are negatively-charged [44, 45]. Given the protective function of the mucus layer in the intestinal tract [46], the presence of this layer in the in vitro model likely mitigates some of the toxic effects of the AgNP and silver ions on the underlying cell layer. However, size has also been shown to be an important factor in the ability of nanoparticles to penetrate mucus, with smaller particles more likely to avoid mucoadhesion [44]. This phenomenon may help to explain the size-specific response seen in this study, in which exposure to 10 nm AgNP caused increased epithelial permeability while exposure to larger particles had no effect. This was supported by our observations, as T84 monolayers treated with 10 nm AgNP showed fewer, lighter cell surface agglomerates than those treated with 20 nm AgNP (data not shown), suggesting that more of the 10 nm particles may have been passing through the mucus layer to the epithelial surface. Regardless, it is clear that the presence of the mucus layer and its impact on AgNP exposure would more accurately reflect an in vivo exposure situation.

One of the most challenging aspects of research in this field is predicting the behavior, dispersity, and integrity of AgNP in the actual gut environment. Numerous studies have shown that AgNP agglomerate and incur other physical changes in culture media and many have assumed that passage of nanoparticles through the GI tract would cause similar effects. However, a study published by Bohmert and colleagues found that processing of AgNP in an artificial digestion simulation did not significantly affect cytotoxicity and only caused minor agglomeration of particles [38]. Other studies have suggested that AgNP may dissolve in the human gut environment, or possibly dissolve and then precipitate as new Ag-based particles [47]. The U.S. EPA currently does not permit the sale of unregistered and untested food contact materials containing nanoparticles [48], but their use is allowed for some other items and household goods [49]. However, AgNP-containing medical products, food contact materials, and supplements continue to be available in many countries and, given that increased intestinal permeability has been linked to numerous health conditions, it is clear that this topic deserves further study. Future work in this area should continue to investigate the impacts of AgNP on permeability, the behavior of AgNP in the gut environment, and include studies that evaluate the effects of chronic, low-dose exposure on intestinal barrier function.

## Conclusions

**Fig. 8:**
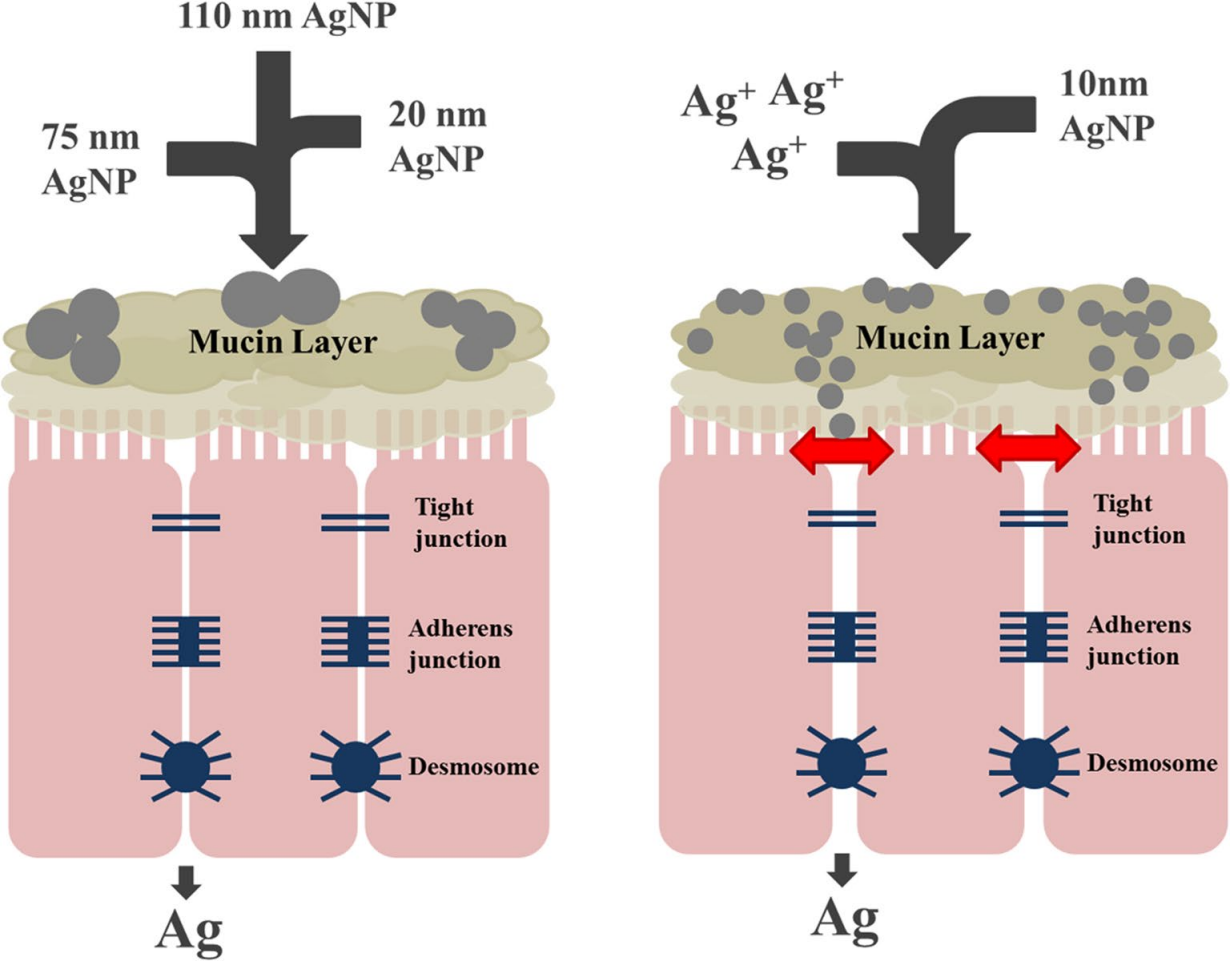
Summary of AgNP effects observed in this study. Exposure to 10 nm AgNP was shown to increase permeability of the epithelial barrier, while exposure to larger particles (20, 75, 110 nm) did not demonstrate any significant effects

This study used a comprehensive approach to evaluate the effects of exposure to AgNP of different sizes and doses on intestinal permeability in an in vitro model of the human gut epithelium. The in vitro system used in this study, T84 human colonic epithelial cells, serve as a superior model for the human epithelial barrier because of their ability to form polarized monolayers and their natural production of a mucus layer. In particular, the presence of this adherent mucus layer allows this model to more accurately predict epithelial-nanoparticle interactions in an in vivo gut environment. Exposure to 10 nm AgNP resulted in increased intestinal permeability at both low (20 µg/mL) and high (100 µg/mL) doses, although effects of the higher dose were observed earlier and were significantly more pronounced. Unlike the elevated permeability caused by treatment with the silver acetate controls, this increase appeared to be independent of any cytotoxicity effects. Comparable effects were not observed for the 20, 75 or 110 nm AgNP at either doses. The observation of nanoparticle-like silver bodies within cells and the detection of silver in basal transwell compartments indicate that both penetration of AgNP into cells and passage of AgNP or derivatives through the cell layer are occurring. Exposure to 10 nm AgNP resulted in the up-regulation of genes involved in the function of tight junctions and desmosomes, possibly indicating that cells were attempting to compensate for the increased permeability by increasing the expression of genes involved in maintaining cell–cell adhesion. The ability of the 10 nm AgNP to cause changes in permeability and gene expression not observed with the larger particles could be due to an increased capacity to transit the protective mucus layer and penetrate the intestinal epithelium (Fig. 8). The results of this study suggest that gastrointestinal exposure to small-size silver nanoparticles may cause increased intestinal permeability, even in the absence of any toxicity effects on cells. This has the potential to cause unwanted effects such as elevated gut inflammation and the increased passage of antigens and microorganisms into circulation.

## Methods

### Silver nanoparticles and nanoparticle characterization

All AgNP were obtained from NanoComposix (San Diego, CA, USA) in BioPure formulations at sizes 10, 20, 75 and 110 nm. Three of the AgNP used in this study (10, 75 and 110 nm) were used in previous work published by this group. In depth characterization of this batch of AgNP, including DLS, TEM, ICP-MS, and zeta-potential analysis was performed on these particles prior to use and characterization data is available in the referenced publications [32, 35]. The 20 nm AgNP was obtained in identical formulation to above particles and in-depth characterization data, including analysis of size [TEM and dynamic light scattering (DLS)], concentration, and zeta potential, was provided by the company. All AgNP were spherical in shape and stabilized with a sodium citrate surface and were suspended in 2 mM sodium citrate solution. Nanoparticles (100 µL of 1 mg/mL solutions) were plated onto Sheep’s Blood Agar plates and incubated for 72 h at 37 °C to confirm sterility. Nanoparticles were also tested using the ToxinSensor Chromogenic LAL Endotoxin Assay Kit (GenScript, Piscataway, NJ, USA) according to the manufacturer’s protocol to confirm lack of endotoxin contamination. Protein corona formation was tested for 20 and 100 µg/mL concentrations of nanoparticles using the protocol described earlier [50]. Briefly, AgNP were incubated for 24 h in T84 complete cell culture medium and water, after which samples were removed and centrifuged at 13,000 rpm for 15 min. AgNP were washed 1X with PBS and resuspended in 30 µL sterile, distilled water. Novex Tris–Glycine SDS Sample Buffer (Life Technologies, Carlsbad, CA, USA) was added to suspensions and samples were boiled at 95 °C for 10 min. AgNP samples and protein ladder were loaded onto 12 % Tris–HCl and 4–20 % Tris–HCl precast gels (Bio-Rad, Hercules, CA, USA) and separated using SDS-PAGE. Finally, gels were stained with Coomassie Blue dye and visualized. Stock solutions of nanoparticles were stored in the dark at 4 °C for the duration of the experiment.

### Cell culture

T84 cells (ATCC CCL-248™), a human colorectal carcinoma cell line, were obtained from ATCC (Manassas, VA, USA). Complete growth media was composed of Dulbecco’s Modified Eagle Medium (DMEM)/F-12 media supplemented with l-glutamine and HEPES (ATCC, Manassas, VA, USA), with added 5 % Fetal Bovine Serum (FBS), Penicillin/Streptomycin, and Fungizone. Cells were grown in complete media using 75 cm^2^ cell culture flasks until approximately 70–80 % confluent and then split using 0.25 % Trypsin–EDTA into desired vessels. Cell cultures were maintained in a 37 °C incubator with 5 % CO_2_ and 95 % humidity.

### Cellular toxicity/viability assays

Cellular toxicity was evaluated by acridine orange/ethidium bromide staining. T84 cells were seeded onto plastic coverslips and allowed to grow as described in the previous section. Cell monolayers were then treated with the AgNP (100 µg/mL dose) for 48 h. Wells treated with sterile, endotoxin-free water and silver acetate (20 and 100 µg/mL) were used as controls. Because of silver acetate toxicity, one well was also treated with 100 µg/mL of silver acetate for 3 h to serve as comparison. After the designated treatment time, AgNP and media were removed from wells and cell monolayers were washed twice with room-temperature Hank’s balanced salt solution (HBSS). Fresh media was then replaced into all wells and acridine orange and ethidium bromide were added to each well (3 µg/mL final concentration for each). The plate was incubated at 37 °C for 15 min, after which coverslips were removed from the plate and visualized on microscope slides using an inverted fluorescence microscope (Life Technologies, Carlsbad, CA, USA). Cellular viability was assayed using the CellTiter-Glo Luminescent Cell Viability Assay (Promega, Madison, WI, USA). Briefly, T84 cells were seeded onto 96-well plates at a density of approximately 5 × 10^4^ cells/well. These cells were allowed to grow until confluent (3–5 days) and then spiked with AgNP and silver acetate (20 and 100 µg/mL). Water-treated cells served as controls and 0.5 % hydrogen peroxide was used as a toxicity control. After treatment for 48 h, media containing AgNP was removed and fresh media was added. The CellTiter-Glo assay was then performed using the manufacturer’s instructions, with the luminescence reading taken using a Cytation 3 Multi-Mode Reader (Bio-Tek, Winooski, VT, USA).

### Transepithelial electrical resistance

Seeding of cells and experimental measurement of transepithelial electrical resistance (TER) was performed according to methods established earlier [26, 51–54]. T84 cells were plated onto 6.5 mm, PFTE, collagen-coated transwell inserts (Corning, Corning, NY, USA) at a concentration of 2.0 × 10^5^ cells/well. Complete cell culture media was added to apical and basal reservoirs and cell monolayers were allowed to grow for 5–10 days, with periodic readings of TER taken using a STX electrode probe and EVOM2 Epithelial Voltohmmeter (World Precision Instruments, Sarasota, FL, USA). Once wells reached approximately 800–1000 Ω, media was changed and cells were allowed to equilibrate for 3 h. A baseline TER reading was then taken, after which nanoparticles or controls were added. AgNP and silver acetate were added to wells for a final concentration of 20 and 100 µg/mL. This range covers the concentration present in consumer use products as well as a high dose (100 µg/mL) to establish a reference/positive control. In a separate well, ethylene glycol tetraacetic acid (EGTA) at a final concentration of 2.5 mM was used as a positive control to induce increased permeability in transwells [40]. TER measurements of cells were taken before exposure to AgNP/controls, as well as 15 min, 1, 2, 3, 24 and 48 h post-exposure. Statistical data analysis was performed using SigmaPlot software (Systat Software, San Jose, CA, USA) and TER measurements are presented as percent of original baseline value (measured before addition of AgNP/controls). After completion of TER transwell studies, media from basal compartments was collected and stored at −20 °C until analysis by inductively coupled plasma mass spectrometry (ICP-MS) could be completed to estimate passage of silver through the cell layer. Given that there are many more 10 nm AgNP than 75 or 110 nm AgNP in the same concentration of (100 µg/mL) AgNP solution, work was also done to establish whether particle numbers of AgNP played some role in the demonstrated effect on permeability. In the TER experiment, differences in permeability were still observed when the number of 10 nm AgNP was adjusted to be roughly that of 110 nm AgNP at 100 µg/mL concentration, suggesting that effects are independent of particle number (data not shown).

### Transmission electron microscopy

For imaging by transmission electron microscopy (TEM), transwell inserts of T84 cells were grown as described previously and spiked with AgNP and controls. After the 48 h time point, all cell culture media was removed and transwells were fixed in 4 % glutaraldehyde (Electron Microscopy Sciences, Hatfield, PA, USA). Fixed tissues were further processed as described earlier [55]. Transwells were imaged at the FDA NanoCore Facility (Jefferson, AR, USA) and energy-dispersive X-ray spectroscopy (EDS) analysis was performed to confirm the presence of silver within cells.

### Immunofluorescence

T84 cells were seeded onto plastic coverslips (Thermo Fisher, Waltham, MA, USA) in a 24-well plate at a density of approximately 2.0 × 10^5^ cells/well and incubated 3–7 days, until cells were found to be confluent by visual observation. Cell monolayers were then treated with 100 µg/mL of the 10 nm AgNP for 24 h. Wells treated with EGTA (2.5 mM), silver acetate (50 µg/mL), and an untreated well were used as controls. After completion of treatment, cell culture media was removed from wells and cells were washed twice with ice-cold PBS. Cells were fixed in a 1:1 mix of methanol and acetone for 15 min at room temperature. Cells were rinsed again with PBS and permeabilized for 3 min with 0.1 % Triton X-100 in PBS. Next, cells were washed twice with PBS and blocked in 3 % milk powder for 1 h at room temperature. Cells were washed again (2 × 5 min) and incubated with a mouse monoclonal primary antibody (15 µg/mL) overnight at 4 °C. Primary antibodies for Occludin and E-cadherin were obtained from the Tight Junction Antibody Sampler Pack (Invitrogen, Carlsbad, CA, USA). Cells were washed in PBS (4 × 5 min) and 5 µg/mL of a fluorescein-conjugated goat anti-mouse secondary antibody solution was added and incubated for 1 h at room temperature, after which cells were washed again in PBS (4 × 5 min). Finally, coverslips were mounted onto microscope slides and visualized using an inverted fluorescence microscope (Life Technologies, Carlsbad, CA, USA).

### QPCR analysis of cellular permeability

RNA was extracted from transwells using the PARIS kit (Life Technologies, Carlsbad, CA, USA) according to the manufacturer’s instructions. Extracted RNA was treated with the Turbo DNA-free kit (Life Technologies, Carlsbad, CA, USA) to remove contaminating DNA and samples were stored at −80 °C until use. The concentration of RNA was quantified using a NanoDrop^®^ ND-1000 (NanoDrop, Wilmington, DE). RNA was reverse transcribed to make cDNA using the Two-Step Reverse Transcription TaqMan kit (Applied Biosystems, Foster City, CA, USA). Gene expression analysis of 84 cell junction-related genes was examined using the RT^2^ Profiler PCR Array Human Cell Junction Pathway Finder (Qiagen, Valencia, CA, USA). Plates were analyzed using an ABI 7500 Real-Time PCR system (Life Technologies, Carlsbad, CA, USA), with amplification conducted with an initial 10-min step at 95 °C followed by 40 cycles of 95 °C for 15 s and 60 °C for 1 min. One independent sample was used for each plate and 3 plates were run for every experimental group. Data was analyzed using the web-based Qiagen Data Analysis Center with normalization of the raw data to provided housekeeping genes (β-ACT, β2 M, GAPDH, HPRT1, and RPLP0). Data from T84 cells treated with 100 µg/mL AgNP (10 and 20 nm) and silver acetate (20 µg/mL) were compared to control cells treated only with UltraPure water (Life Technologies, Carlsbad, CA, USA). Changes in gene expression for RT^2^ Profiler plates were evaluated by Student’s t-test, with genes with p < 0.05 designated as significant.

## Additional file

10.1186/s12951-016-0214-9 Energy Dispersive Spectroscopy (EDS) spectra for transmission electron microscopy (TEM) images of T84 cells. **Figure S2.** Expression of E-cadherin protein in T84 cells. **Figure S3.** T84 cell monolayer after Periodic acid-Schiff staining, showing presence of mucus layer. **Table S1.** Percentage of total spiked silver detected in basal compartment of T84 transwells after treatment with AgNP and silver acetate, as measured by ICP-MS.

## Abbreviations

AgNP: silver nanoparticles
DLS: dynamic light scattering
EDS: energy dispersive X-ray spectroscopy
EGTA: ethylene glycol tetraacetic acid
GI: gastrointestinal
ICP-MS: inductively coupled plasma mass spectrometry
TEM: transmission electron microscopy
TER: transepithelial electrical resistance
