# Supplementary material for: Size and dose dependent effects of silver nanoparticle exposure on intestinal permeability in an in vitro model of the human gut epithelium
Source: J Nanobiotechnology. 2016 Jul 28;14:62. doi: 10.1186/s12951-016-0214-9 (PMC4963959; doi:10.1186/s12951-016-0214-9)
Supplement: Supplementary file 1 — 10.1186/s12951-016-0214-9 Energy Dispersive Spectroscopy (EDS) spectra for transmission electron microscopy (TEM) images of T84 cells. Figure S2. Expression of E-cadherin protein in T84 cells. Figure S3. T84 cell monolayer after Periodic acid-Schiff staining, showing presence of mucus layer. Table S1. Percentage of total spiked silver detected in basal compartment of T84 transwells after treatment with AgNP and silver acetate, as measured by ICP-MS. [file 12951_2016_214_MOESM1_ESM.doc]

**Additional File 1**

***Size and Dose Dependent Effects of Silver Nanoparticle Exposure on Intestinal Permeability in an In Vitro Model of the Human Gut Epithelium***

**Figures**

**
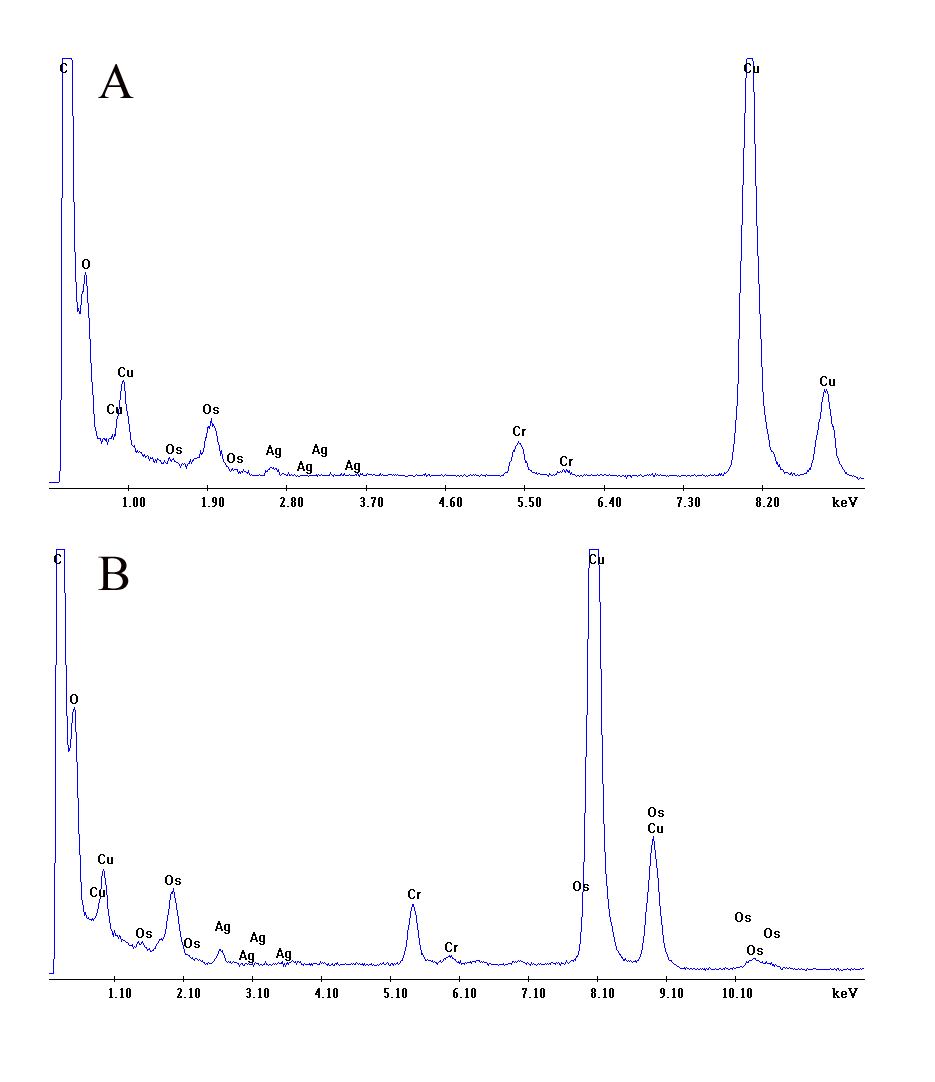
**

**Figure S1.** Energy Dispersive Spectroscopy (EDS) spectra for transmission electron microscopy (TEM) images of T84 cells. (A) Spectrum for image shown in Figure 3A, (B) Spectrum for image shown in Figure 3B.

**
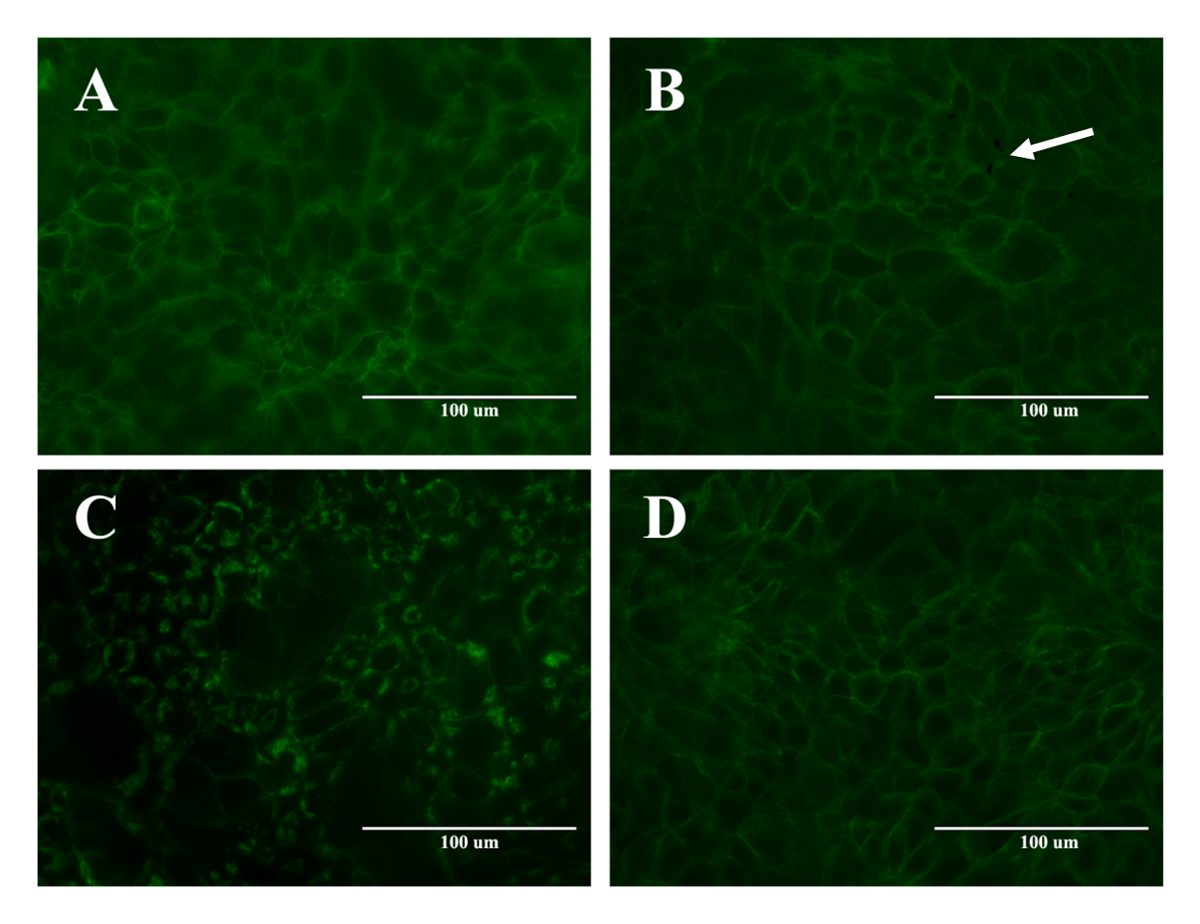
**

**Figure S2.** Expression of E-cadherin protein in T84 cells after 24 hour treatment with (A) control, (B) 10nm AgNP, (C) EGTA, and (D) silver acetate. Arrows point to suspected nanoparticle agglomerates in AgNP-treated cells.


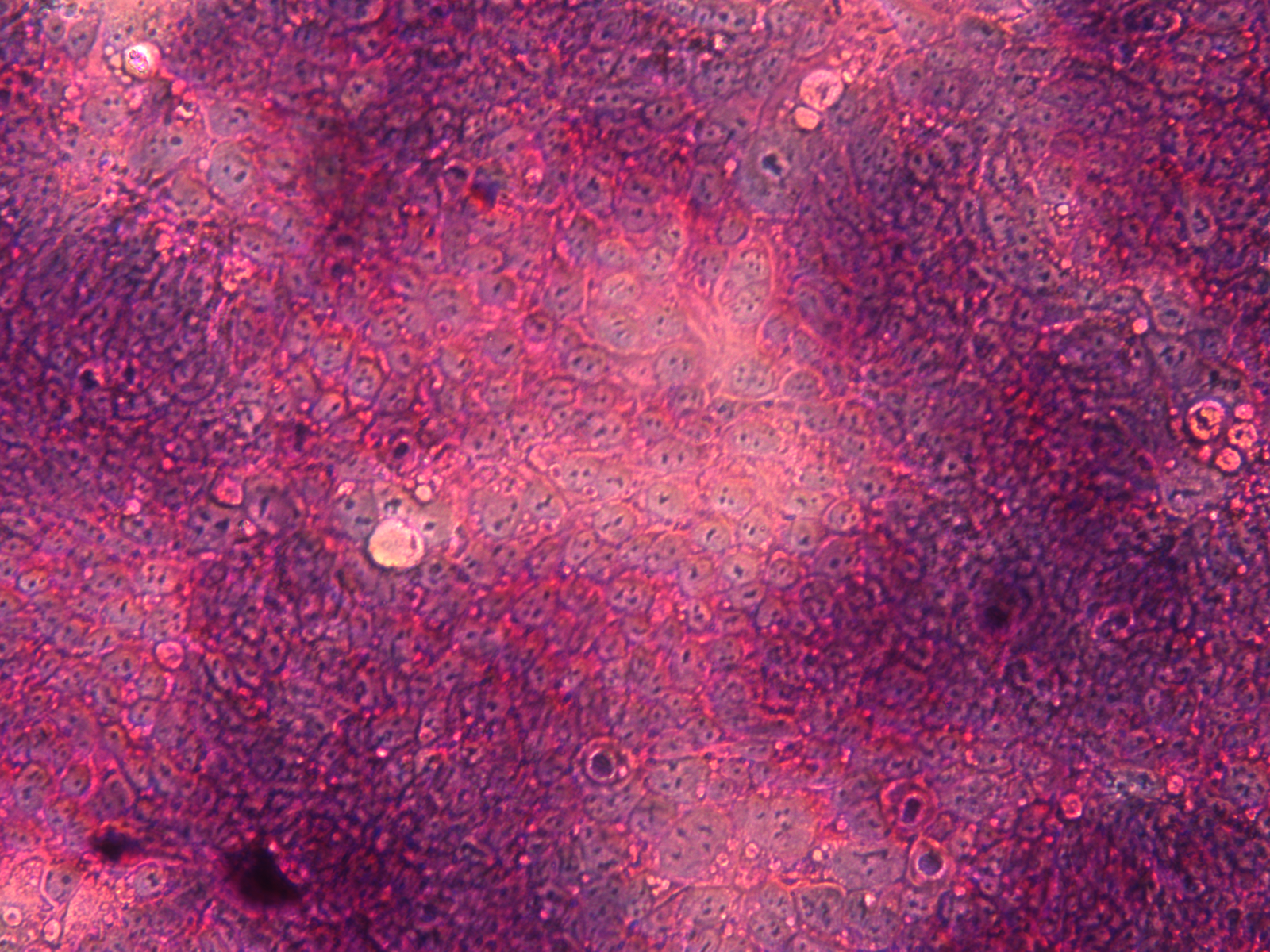


**Figure S3.** T84 cell monolayer after Periodic acid-Schiff staining, showing presence of mucus layer (purple)

.

**Tables**

**Table S1.** Percentage of total spiked silver detected in basal compartment of T84 transwells after treatment with AgNP and silver acetate, as measured by ICP-MS.

| **Time Point** | **Dose** | **10nm** | **20nm** | **75nm** | **110nm** | **Silver Acetate** |
| --- | --- | --- | --- | --- | --- | --- |
| 2 hours | 20ug/mL | BD | BD | BD | BD | BD |
|  | 100 ug/mL | BD | 0.90% | 1.44% | 0.82% | BD |
| 48 hours | 20ug/mL | BD | BD | BD | BD | BD |
|  | 100 ug/mL | 0.72% | BD | 0.71% | 4.19% | 6.06% |

BD: Below Detection Limits (<5 ng/mL).
